# Bubble-enhanced basanite–tephrite mixing in the early stages of the Cumbre Vieja 2021 eruption, La Palma, Canary Islands

**DOI:** 10.1038/s41598-023-41595-3

**Published:** 2023-09-08

**Authors:** Diego González-García, Thomas Boulesteix, Andreas Klügel, François Holtz

**Affiliations:** 1 Institut für Mineralogie, Leibniz Universität Hannover, Hannover, Germany; 2 Volcanology Research Group, Instituto de Productos Naturales y Agrobiología (IPNA-CSIC), La Laguna, Tenerife, Spain; 3 Fachbereich Geowissenschaften, Universität Bremen, Bremen, Germany

**Keywords:** Geochemistry, Petrology, Volcanology, Natural hazards

## Abstract

Syneruptive magma mixing is widespread in volcanic eruptions, affecting explosivity and composition of products, but its evidence in basaltic systems is usually cryptic. Here we report direct evidence of mixing between basanitic and tephritic magmas in the first days of the 2021 Tajogaite eruption of Cumbre Vieja, La Palma. Groundmass glass in tephritic tephra from the fifth day of the eruption is locally inhomogeneous, showing micron-scale filamentary structures of Si-poor and Fe-, Mg-rich melt, forming complex filaments attached to bubbles. Their compositional distribution attests the presence of primitive basanitic magma, with compositions similar to late-erupted melts, interacting with an evolved tephritic melt during the first week of the event. From filament morphology, we suggest their generation by dragging and folding of basanitic melt during bubble migration through melt interfaces. Semi-quantitative diffusion modelling indicates that the filamentary structures are short-lived, dissipating in timescales of tens of seconds. In combination with thermobarometric constraints, we suggest a mixing onset by sub-Moho remobilization of a tephritic reservoir by basanite input, followed by turbulent ascent of a mingled magma. In the shallow conduit or lava fountain, bubble nucleation and migration triggered further mingling of the distinct melt-phases. This phenomenon might have enhanced the explosive behaviour of the eruption in such period, where violent strombolian explosions were common.

## Introduction

Ocean islands, i.e., large intraplate volcanic edifices constructed over thin ocean crust, are characterized by a wide range of eruptive styles, magma compositions and edifice morphologies. A common feature among them is a multi-level plumbing system spanning from the deep mantle to shallow reservoirs in the island edifice^1–6^. In this architecture, interactions between magmas stored at different levels of the system are common and are most conspicuously recorded in bimodal mafic-felsic systems by e.g., the occurrence of banded pumices^7–9^. In basaltic systems, the evidence of magma mixing is commonly cryptic due to the compositional similarity of end-members, high temperature, and low viscosity contrast, making them more susceptible to quick homogenization^10^. In such scenarios, mixing and recharge are usually preserved in the form of zoned minerals^11–16^, and the presence of compositionally heterogeneous products is rarely observed^17^. Mafic recharge, either in bimodal or dominantly basaltic systems, is regarded as a potential eruption-triggering mechanism and is capable of enhancing the explosive potential of an eruption^18,19^.

With the increase of volcano monitoring resources, including near real-time petrologic monitoring, high-resolution temporal datasets have become available. Although temporal variations in bulk rock chemical composition are common in long-lasting basaltic eruptions, their importance has been highlighted in some recent events (e.g., 2021 Fagradalsfjall eruption, Iceland^20,21^ or the 2018 Kilauea Lower East Rift Zone eruption^22,23^).

The highly destructive Cumbre Vieja 2021 eruption in La Palma, Canary Islands, was an exceptionally well monitored and sampled monogenetic eruption, fed by ocean island basalt (OIB) magma. Temporal variations in magma chemistry were recorded, suggesting magma mixing before and during eruption^24^. Here we present major element compositions of groundmass glass in tephra, crystallized groundmass from lava, and minerals from both tephra and lava from the eruption to shed light on magma dynamics and interactions occurring in its early phase, where a diverse array of lithologies and mineral cargoes were erupted. Particularly, we focus on conspicuous heterogeneities in tephra glasses from the fifth day of the eruption, i.e., 23 September 2021, with complex filamentary structures associated to bubbles. This tephra was erupted during strongly enhanced explosive activity in the Tajogaite cone. We combine chemistry and diffusion modelling in the filamentary areas with thermobarometric estimations to draw a picture of magma mixing and ascent dynamics taking place in the first days of the eruption. These data provide exceptional direct evidence of melt-melt interactions in a basaltic system and, complementing mineral studies, represents a window to magmatic processes occurring at different depths and feeding monogenetic eruptions in ocean island setting.

### The Cumbre Vieja 2021 eruption

**Figure 1:**
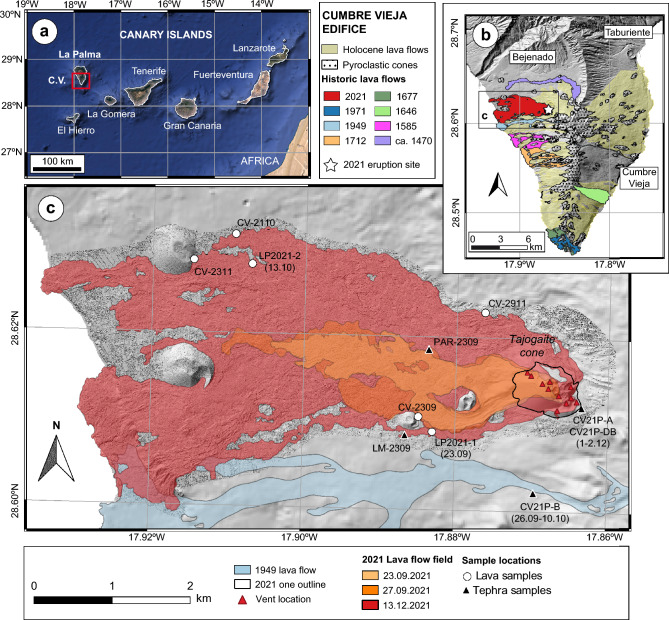
(**a**) Location of La Palma and the Cumbre Vieja (CV) edifice in the Canary Islands. (**b**) Simplified geology of the Cumbre Vieja rift with prehistoric and historic lava flow fields. (**c**) Detail of the 2021 eruption site with sampling locations. Geologic units are from the GRAFCAN data repository, and 2021 lava flow outlines and vent locations are from Copernicus Emergency Management System (European Commission). Sample emplacement date is included in sample name except LP2021 and CV21P samples, where it is given in parenthesis. CV shaded relief base map was calculated from the digital elevation model (DEM) from GRAFCAN, and that of the 2021 lava flow field was obtained from the high-resolution DEM^45^, from which the final cone contour was drawn. These maps were generated using QGIS v. 3.16 (https://qgis.org).

The Canary Islands represent one of the best examples of OIB magmatism connected to a deep-seated mantle plume^25–28^. La Palma and El Hierro are the westernmost and youngest islands of the Canary Islands (Fig. 1), and La Palma’s Cumbre Vieja (CV) ridge is historically the most volcanically active edifice of the whole archipelago. CV registered a total of eight confirmed eruptions since late fifteenth century, with recurrence periods between 22 and 237 years. The geochemistry of La Palma lavas defines a highly alkaline magma series spanning from basanite to phonolite but is volumetrically dominated by basanites and tephrites^29^. Phonolites are almost absent in the older Taburiente and Bejenado edifices but are common on CV^29^, where juvenile phonolite was involved in the 1585 Jedey eruption^30^. Mixing between phonolite and more primitive magmas has been recently documented^31^. From petrological studies, several authors have revealed a plumbing system structured in three distinct storage regions at 30–35 km, 18–28 km and a shallow, intracrustal one at 7–14 km^32–34^, which are in broad agreement with the main locus of recent seismic activity under the island^35–38^.

The CV 2021 eruption (September 19–December 13, 2021) is one of the best monitored basaltic monogenetic eruptions in an ocean island setting. Low intensity seismic swarms, likely representing the first signs of unrest, were detected in October 2017, February 2018, and July 2020 to February 2021, at depths between 20 and 30 km^35,36,39^. On 11 September 2021, an intense seismic swarm started under the CV rift, with hypocentre depths of < 12 km. The renewed seismic activity was accompanied by edifice inflation^36^, suggesting an increased probability of eruption. Indeed, after only one week of precursor activity, eruptive fissures opened at 14:10 UTC on 19 September at an altitude of ca. 880 m.a.s.l. on the west-facing slope of the CV edifice. During the early phase of the eruption, the activity consisted in intense strombolian explosive activity, alternating lava fountains and rapid strombolian explosions^40^, some of which produced conspicuous atmospheric shock waves on 23–24 September. After an activity hiatus lasting for several hours on 27 September, the eruption resumed with renewed energy, but tephra production diminished progressively. During October and November, the most voluminous lava extrusion phase was dominated by low viscosity lavas^41^, and the emission of lava and pyroclasts occurred in distinct vents^42,43^. However, in the last days of the eruption, explosivity increased again, manifested by a phreatic vulcanian phase with a column height of 8 km^43,44^. The volume of erupted materials was estimated to be 0.217 km^3^ of lava flows and 0.023 km^3^ of tephra, representing the largest erupted volume among historical eruptions in La Palma^43,45^.

Whole rock compositions and mineral contents of the lavas varied significantly during the 85-day eruption. Lava flows erupted during at least the first 9 days of the eruption, until the 27 September pause (Fig. 1c), were tephritic with a high phenocryst content dominated by clinopyroxene and amphibole^46^, with bulk MgO contents of 5–7 wt%. They were followed by a more primitive, olivine-clinopyroxene basanite^24,46^, with MgO content increasing to 8–9 wt%. This tephrite-basanite sequence is analogous to that observed during the multi-vent 1949 eruption^32^ and the 1971 Teneguía eruption^47^. In comparison to 2021, the 1949 event covered a wider compositional range, erupting a more evolved tephrite (MgO 3.3–6.4 wt%) and also minor phonotephrite.

## Results

### Glass and crystallized groundmass chemistry

**Figure 2:**
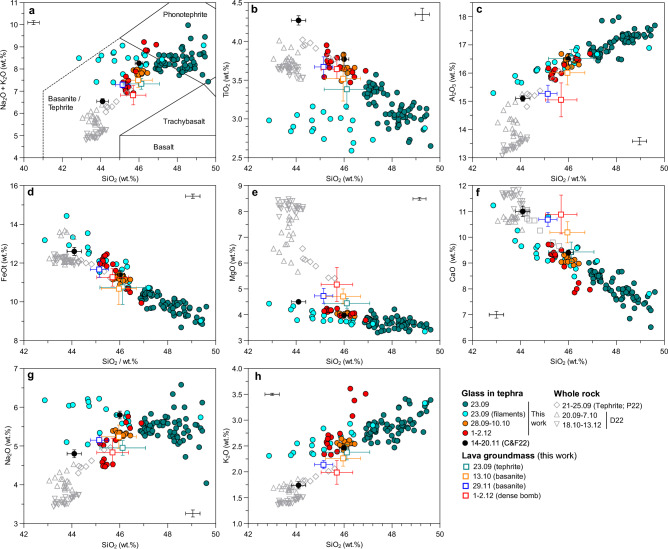
Geochemical distribution of glass in tephra, crystalline groundmass in lava, and lava whole rock data by emplacement date. (**a**) Total alkali vs silica (TAS) diagram, (**b–g**) Harker diagrams. Coloured spots are data measured for this work, and black and grey points represent literature data. Circles represent glass analysis, squares are crystalline groundmass, and triangles are whole rock data. Points measured in the filamentary structures are highlighted in light blue. Literature data from Castro and Feisel^41^ (C & F22), Pankhurst et al.^46^ (P22) and Day et al.^77^ (D22).

Major elements concentrations of groundmass glasses in tephras and lavas are reported in Fig. 2. Lava bulk groundmass was analysed in samples from September 23 (tephrite), October 13, November 29 and a dense bomb from 1–2 December (basanites; see “Methods” section) by laser ablation-inductively coupled plasma-mass spectrometry (LA-ICP-MS). Results indicate compositions intermediate between groundmass glasses from the later stages of the eruption and whole rock data from the literature. They are slightly more enriched in MgO (4.4–4.7 wt%) and CaO (9.4–10.9 wt%) than tephra glasses, and they seem to follow a trend towards whole rock data^24^. The groundmass in the tephrite is the more evolved (4.4 wt% MgO), however falling within error of groundmass glasses in later basanitic samples.

**Figure 3:**
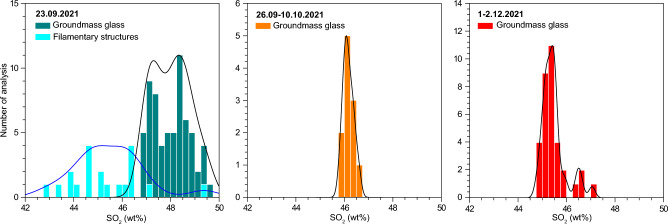
Histograms and probability density distributions, representing the compositional distributions of tephra groundmass glasses (i.e. those far from the influence of crystals or filamentary regions) and filamentary structures.

Groundmass glass analyses in tephras show a continuous trend spanning through the basanite/tephrite to the phonotephrite fields in the TAS diagram. These compositions are more evolved than corresponding whole rock analyses^24,46^ due to the presence of Fe–Mg-bearing phenocrysts, but follow an analogous evolution towards primitive compositions. Glasses erupted on 23 September straddle the fields of tephrite and phonotephrite, while glasses from later in the eruption evolve towards more mafic compositions. The most primitive terms from our samples were erupted on 1–2 December, but glasses erupted between 14 and 21 November^41^ are more primitive than most glasses studied here (Fig. 2). Tephritic glasses from 23 September average ca. 47–49 wt% SiO_2_, 3.3–4.0 wt% MgO, 8–9 wt% alkalis, whilst glasses in the late deposit from December 1–2 show 45–46 wt% SiO_2_, 4.0–4.5 wt% MgO and 7–8 wt% alkalis. The September 26–October 10 glasses are intermediate but closer to the mafic end of the measured compositional spectrum. Tephra groundmass glasses from September 23 have a notably wider compositional range than later glasses. Compositional histograms (Fig. 3) evidence that on 23 September, SiO_2_ varied between 43 and 49 wt%, with two modes at ca. 47 and 48.5 wt%. This contrasts to later samples, with well-defined unimodal distributions centred at 45–46 wt% SiO_2_.

**Figure 4:**
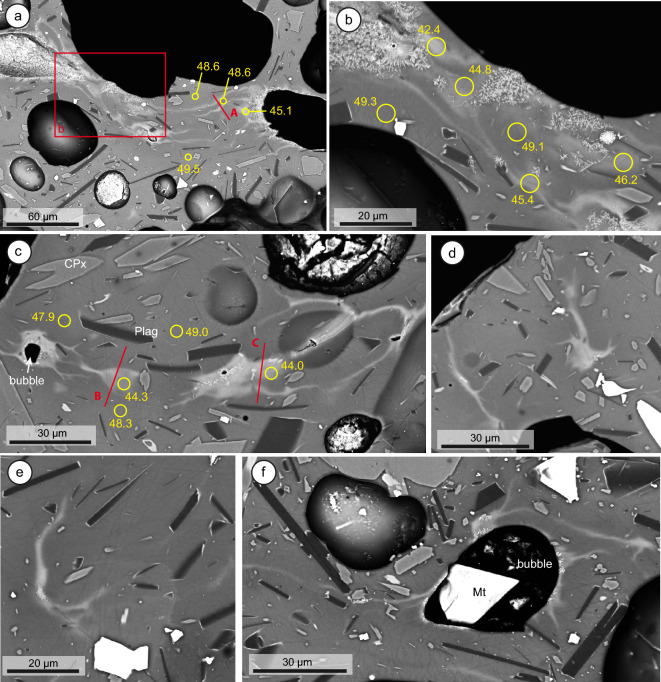
Backscattered electron images of melt mingling features in the El Paraiso lapilli sample collected on 23 September 2021. (**a,b**) Complex filamentary field connecting large bubbles, showing dendritic magnetite growth in the inner parts of mafic filaments and bubble rims. (**c**) Small bubble (left) surrounded by mafic melt; (**d,e**) small, isolated filaments apparently unrelated to bubbles. (**f**) Bubble associated to filaments and magnetite (Mt) crystal, suggesting heterogeneous bubble nucleation. EMPA beam transects (A–C) in Fig. 5 and selected EPMA 5 µm analytical spots are highlighted, with their SiO_2_ concentrations (wt%) attached.

The most remarkable characteristic of 23 September tephra is the presence of conspicuous chemical inhomogeneities at very small scales (Fig. 4). Inhomogeneous melt areas dominated by filamentary, fluidal structures are evident in back-scattered electron (BSE) images. Filament lengths are in the order of 150–300 µm and widths do not exceed 20 µm. Such features are reminiscent of magma mingling structures (i.e., advection or physical mixing), being dominated by stretching and folding of a primitive magma inside a more evolved one^48,49^, and strongly resemble those observed in banded pumices with notably larger scale and compositional contrast^9^. These structures are very often associated to bubbles, with filaments commonly radiating from them, but some of them are detached, likely due to sectioning effects (Fig. 2d,e). The central parts of the filaments and bubble rims associated to them frequently show small, dendritic crystals of a Fe–Ti-rich mafic phase, which is identified as titanomagnetite in SEM–EDX mapping (Supplementary Fig. 5). Electron microprobe (EMPA) analyses (blue dots in Fig. 2) demonstrate that these mixing areas have a wide range in composition, from more primitive glass (44 wt% SiO_2_, 4.5 wt% MgO, 13 wt% FeO, 11 wt% CaO) represented by the light-toned areas and filaments in BSE images, to more evolved, tephritic and phonotephritic-like compositions (49 wt% SiO_2_, 3.5 wt% MgO, 8 wt% FeO, 7 wt% CaO) in the background glass. The primitive melts, which we will refer to as “basanitic”, compositionally overlap with groundmass glasses of tephras erupted during October-December (Fig. 2), indicating that a basanitic magma component was already present at the beginning of the eruption.

Nevertheless, for Na_2_O and TiO_2_, the basanitic melts in the filament areas do not co-vary with Si and deviate from the evolutionary trend of other samples from the eruption, as evidenced by light blue dots in Fig. 2. Na_2_O concentrations are 5.5–6.2 wt%, similar to, or slightly higher than, phonotephritic melts in the most evolved end. Similarly, TiO_2_ shows a restricted concentration range around ca. 3.0 wt%, contrasting with concentrations up to 3.5–4.0 wt% in more primitive groundmass glasses present later in the eruption. The Na_2_O trend likely reflects a homogenization of the filamentary structures due to differential diffusion of Na compared to the remaining major elements. The diffusivity of Na in basaltic melts is up to 2 orders of magnitude larger than that of Si and other major elements^10,50^ (Supplementary Table 2), leading to a significantly faster homogenization at magmatic conditions. In contrast, the relative Ti depletion appears to be related to crystallization of dendritic magnetite in the filaments.

**Figure 5:**
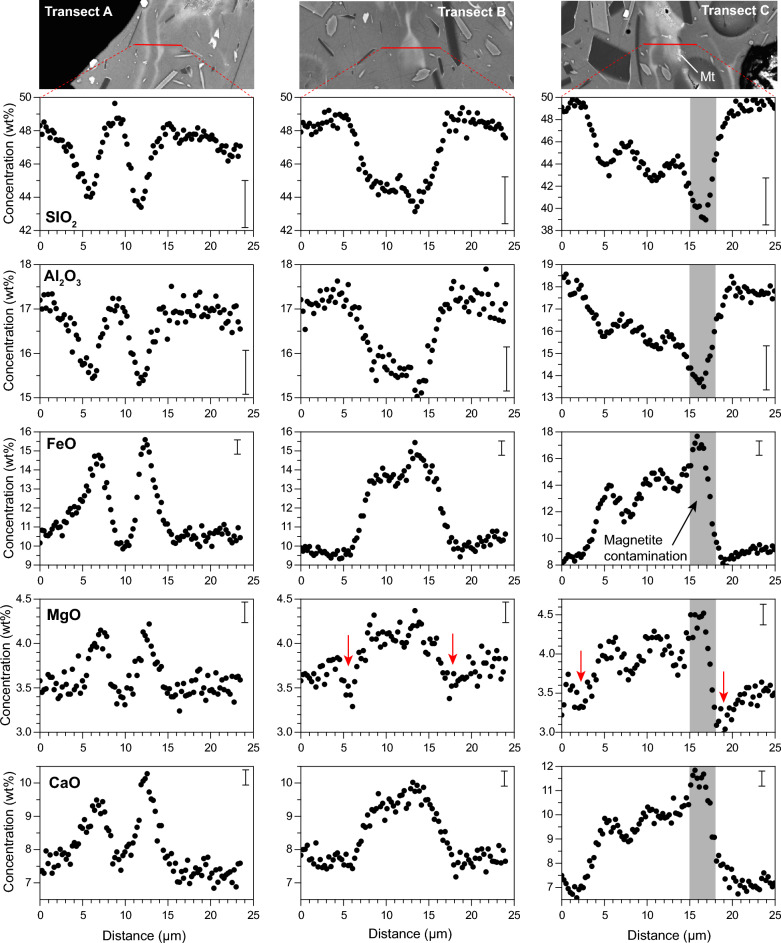
EPMA beam transects across three different filaments in sample PAR-2309, from 23 September 2021. Error bars indicate 2-sigma precision of the semiquantitative analysis. The red arrow in MgO transects shows likely multicomponent diffusion effects (uphill diffusion) resulting in concentration minima at both sides of the basanitic filament. Transect C is likely affected by slight analytical contamination form magnetite dendrites centred at ca. 16 µm (grey band), resulting in a distinct peak and a wider compositional span.

We performed three EMPA high-resolution beam profiles across the most prominent filamentary structures, allowing a semi-quantitative estimation of glass chemistry with a resolution of < 1 µm (see “Methods” section). Results (Fig. 5) show that compositional diversity in the profiles is consistent with the compositional range in EMPA spot analyses, with 44–50 wt%, SiO_2_, 3.5–4.4 wt% MgO and 7–10 wt% CaO. Sigmoidal profiles across the compositional interface suggest diffusive exchange between melts of different composition. The only deviation occurs in profile C, where clear maxima and minima are centred at around 15–17 µm, in which estimated FeO concentrations reach 18 wt%. This is significantly larger than the most iron-rich compositions of groundmass glasses (13 wt%), and it is likely the result of a mild amount of contamination of the analysis by dendritic magnetite crystals commonly occurring in the central part of the mafic filaments. The shape of the profiles is also variable. Transect A cuts across a double, thin filament, and displays two simple compositional peaks/valleys. Transects B and C are more complex, with plateau-shaped bands where one or more peaks can be present and are therefore indicative of complex dynamics^48,49^ leading to their final state.

### Mineral cargo of tephra and lava

Among the sampled materials, two distinct lithologies can be recognized based on the phenocryst cargo in lavas and tephra^24,46^: (1) an amphibole-pyroxene tephrite represented by materials emplaced on 23 September, simultaneously to tephra with inhomogeneous glasses, and (2) an olivine-pyroxene basanite represented by samples emplaced between October and December. The tephritic lava is porphyritic, with a phenocryst population dominated by amphibole and clinopyroxene in a proportion of ca. 1:1, accompanied by < 5% titanomagnetite and rare olivine. Gabbroic cumulate xenoliths composed of olivine, clinopyroxene and magnetite are common in the tephritic lavas (Supplementary Fig. 1). Amphiboles in the lava flows are frequently resorbed whilst those in tephra are euhedral in shape. Plagioclase laths, with an An_58–67_ composition^46^, are common both in lava groundmass and tephra along with amphibole, olivine, clinopyroxene and apatite. Scarce sulphide melt blebs are also present. In contrast, later basanites show a lower phenocryst population composed of clinopyroxene and olivine in proportions of 2:1, with minor titanomagnetite. Clinopyroxenes show complex zonings and abundant melt inclusions in both cores and mantles. The groundmass fraction is formed by clinopyroxene, olivine, plagioclase, apatite and magnetite.

EMPA analyses (Supplementary Information) show that pyroxene phenocrysts in both lavas and tephra are diopsides. Complex sector and/or concentric zonings are common, and a subset of pyroxenes from tephritic samples show heavily resorbed cores. Melt inclusions are widespread. Rims follow linear relationships between Mg# and Al_2_O_3_, TiO_2_, SiO_2_ and FeOt, for Mg# between 67 and 79. The effects of sector zoning^51^ in rims are evident, with two compositionally distinct populations (Supplementary Figs. 2, 3). Low-Al, hourglass sectors have Al_2_O_3_ < 6.5 wt%, low Na_2_O (< 0.5 wt%), high SiO_2_ (46–50 wt%) and high Mg# (73–80). In contrast, high-Al prism sectors are characterized by Al_2_O_3_ > 6.5 wt%, Na_2_O of 0.5–0.8 wt%, SiO_2_ between 40 and 47 wt% and Mg# of 67–77. Cores and mantles show a wider compositional variety than rims, with Mg# in the range 52–82 and non-linear relationships between Mg# and most compositional parameters. The effects of sector zoning in cores appears to be minimal. Cores with Mg# < 65 seem to be almost exclusive to tephrites from 23 September.

Amphiboles in the tephritic lava and tephra are classified as kaersutites. Mineral formula calculated using the AMFORM mass-balance model^52^ results in Mg# 55–71 and Si 5.7–5.9 atoms per formula unit (apfu). Kaersutites are commonly zoned, with cores showing more restricted Mg# (55–67) and lower SiO_2_ (38–40 wt%), CaO (11.4–12.2 wt%) and TiO_2_ (5.3–5.7 wt%) than rims (Supplementary Fig. 4).

Olivine is a major phenocryst phase in lavas and tephra emplaced during October to December, although they also appear in low proportions in the groundmass fraction in lavas and tephras from 23 September. Core compositions are in the range Fo_78-84_, with NiO contents ranging from 0.10 to 0.26 wt% for most late olivines, whereas two olivine analyses from tephrite samples display values of 0.07 to 0.08 wt% NiO.

### Storage conditions of endmembers

**Figure 6:**
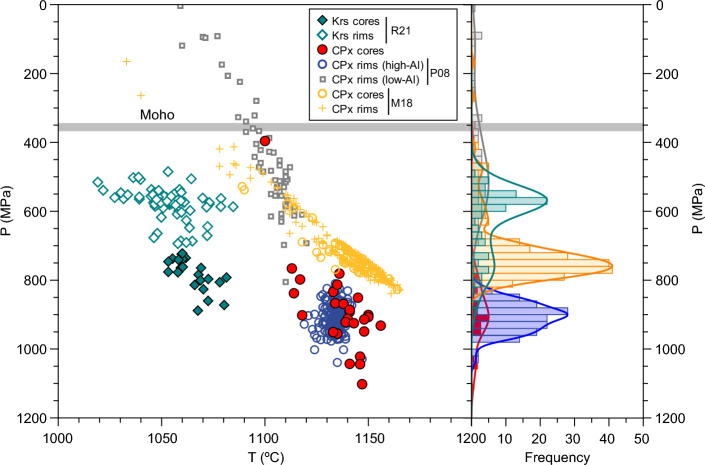
Summary of thermobarometric results in the CV 2021 eruption products, including pressure distribution and kernel density estimates (right). Kaersutite (Krs) thermobarometry was calculated with the Ridolfi^61^ calibration (R21), and those of clinopyroxene (CPx) were obtained using the Putirka^59^ and Mollo et al.^60^ calibrations (P08 and M18, respectively). Moho pressure (ca. 350 MPa) derived from estimated Moho depth of 12.5 km below La Palma^83^.

Using our mineral and groundmass glass data, we applied various geothermometers and geobarometers to obtain information on storage regions of magmas involved in the eruption. Results are summarized in Fig. 6 and Supplementary Table 1. Clinopyroxene is well suited for thermobarometry, but a major difficulty arises from the effects of sector-zoning^51,53–55^. Under variable undercooling conditions, crystals develop prism sectors {h, k, 0} and hourglass sectors {− 1, 1, 1} reflecting disequilibrium during rapid growth. Under low undercooling, hourglass sectors are enriched in Mg and Si, and depleted in Al and Ti, relative to prism sectors^51,56^. This observation hampers the appropriate choice of sector for thermobarometric evaluation. Although the effects of sector zoning on thermobarometric results are not yet well established^51,53,54,57,58^, low undercooling apparently does not produce large discrepancies between sectors^51^. Other works^56,57^ favour low-Al sectors as best recorders of equilibrium conditions. Since the effects of sector zoning are readily visible in the CV 2021 pyroxene rim data (Supplementary Fig. 3), in the present work we treat separately high-Al sectors (Al_2_O_3_ > 6.5 wt%) and low-Al sectors (Al_2_O_3_ < 6.5 wt%) and briefly discuss the significance of their thermobarometric results.

Results are shown in Fig. 6 and Supplementary Table 1, and the complete dataset of calculated conditions is available in the Supplementary Information. For clinopyroxene rim compositions, the iterative procedure outlined in the Methods section combined with the Putirka^59^ equations discards low-Al rims, reducing the scatter of results. In consequence, the rim data cluster around a temperature of 1140 °C and a pressure of 800–1000 MPa. However, when low-Al sectors are treated separately using the same procedure, they define a continuous array with a temperature range of 1060–1120 °C and pressure range of 0–800 MPa, notably lower than high-Al sectors. Data of clinopyroxene core analyses overlap completely with high-Al rim data and cluster around 1140 °C and 900 MPa, albeit with larger scatter; a few analyses produce shallower results. Compared to the Putirka^59^ calibration, the calibration for mafic alkaline magmas (MAM) of Mollo et al.^60^ is less efficient in filtering out low-Al data, producing a more continuous P–T array. Calculated temperatures are comparable to the Putirka calibration, but the pressure range is 400–800 MPa with most values in the range 650–800 MPa. In our dataset, the clustering of the high-Al rim data and the agreement between pressures obtained for high-Al rims and clinopyroxene cores, argue in favour of high-Al data as recorders of storage conditions. Low-Al sectors would in turn reflect decompression during the last stage of growth at high undercooling degrees. In consequence, we favour a pressure of 800–1000 MPa for clinopyroxene storage, as recorded by high-Al sectors, with an alternative value of 650–800 MPa as provided by the MAM calibration^60^. The comparison of pressure values derived from tephrites and basanites yield no significant differences.

Storage conditions were evaluated in kaersutites using a mineral-only calibration^61,62^. Results show a bimodal distribution of P–T conditions. Rims and mantles (n = 68) of kaersutites from both tephras and lavas indicate a pressure interval of 485 to 687 MPa, with an average and standard deviation of 580 ± 42 MPa; and a temperature of 1019–1085 °C, with an average of 1052 ± 13 °C. In contrast, a group of core analyses yield a higher pressure of 722–888 MPa and temperatures of 1053–1081 °C (with averages of 783 ± 46 MPa and 1065 ± 8 °C). These results were cross-checked with an amphibole-melt barometer^63^, resulting in broadly comparable or slightly larger pressures. The presence of amphibole and the lack of plagioclase phenocrysts in the early-stage eruption products argue in favour of a melt with H_2_O contents in the order of 3 wt%, as suggested by experimental constraints on the amphibole stability field in basanitic magmas^64^.

## Discussion

### Lifespan of heterogeneous glasses

The compositional gradients across the observed filamentary structures (Fig. 5) are reminiscent of diffusive element exchange between distinct melts, hence a diffusion-based estimation of the interaction timescales between both melts is possible. Contrary to minerals, diffusion timescales in melts are notably more complicated to obtain since diffusion (chemical mixing) is superimposed to advection during the mingling of melts. Advection, or physical mingling, contributes to the mixing process by providing extensive contact area between the melts^48,49,65,66^, and thus enhancing their homogenisation rate.

We have modelled the expected survival time of the filaments by means of a finite difference diffusion model of the SiO_2_, Al_2_O_3_ and CaO profiles, without considering the effects of advection (see “Methods” section). The choice of an initial concentration gradient is a key step in diffusion modelling. In our case, due to the dynamic nature of a melt system, such a choice would be highly speculative, especially in the more complex profiles. Instead, we have opted for using the measured profiles as starting point, with the aim of assessing their homogenization times. The normalized concentration variance^67^ of each element (${\sigma }_{n}^{2}$; i.e., the ratio of initial to modelled concentration variance across the profile) was calculated in each step to monitor the degree of chemical homogenization.

**Figure 7:**
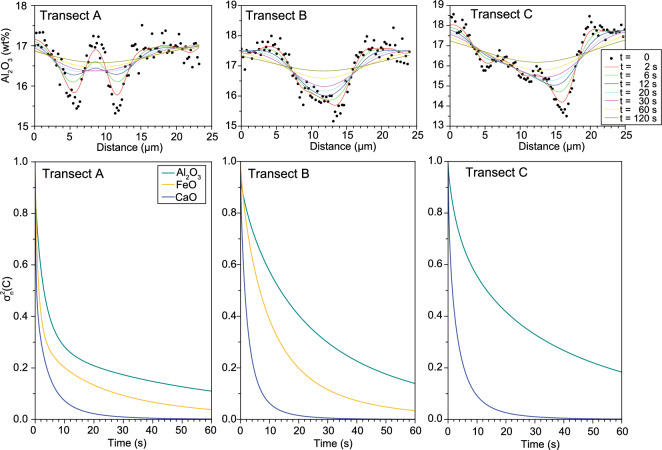
Top row: results of the finite difference diffusion model for the Al_2_O_3_ profiles, in which the measured beam profiles are used as starting point. Bottom row: Evolution of normalized concentration variance, σ_n_^2^(C), of the three beam profiles for Al_2_O_3_, FeO and CaO. In Transect C, FeO data was discarded due to analytical contamination from magnetite crystals.

**Figure 8:**
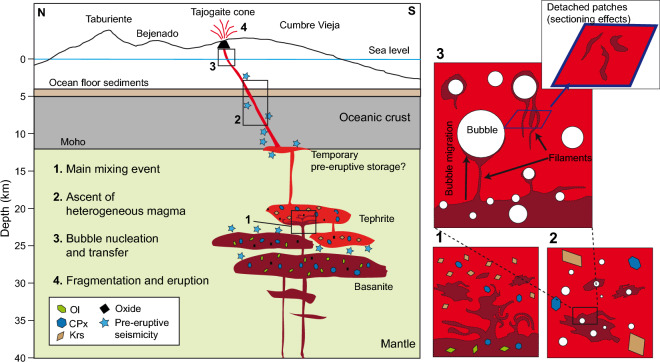
Sketch of magma storage and ascent during the early stage of CV 2021 eruption (not to scale). A storage level of 25–35 km is envisaged for basanitic magmas, which could be responsible for pre-eruption seismicity. Crystal-rich tephritic magmas could instead have evolved in a shallower reservoir at 16–23 km, which became intruded (1) by the basanitic magma on early September 2021. Subsequently, a heterogeneous magma quickly ascended through the ocean crust (2) and island edifice, until major bubble nucleation at level (3) further enhanced mixing and likely influenced eruption explosivity.

The calculated evolution of the concentration profiles with time is shown in Fig. 7, and the dataset is provided in the Supplementary Information. The compositional gradients in all three profiles significantly decrease within a few seconds, decaying exponentially with time^67^. The value of ${\sigma }_{n}^{2}$ diminishes by 20% to 50% after only 5 s, depending on the diffusion rate of each element and the initial thickness of the filaments. After 1 min, the concentrations gradients mostly disappear, reaching levels of < 20% of the initial ${\sigma }_{n}^{2}$. By inference, the diffusive exchange from the initial to the measured concentration profile must have occurred in a similar timescale. These extremely short timescales of survival are too small to represent primary mixing processes occurring at depth, differing by orders of magnitude to those previously determined for other volcanic events using chemical heterogeneities in melts^7,68,69^. Complete homogenization (${\sigma }_{n}^{2}$ < 0.01) would be reached in timescales varying from 54 to 76 s (Ca) to 594–836 s (Al), depending on element diffusivity. We conclude that the observed filamentary structures are short-lived and were therefore produced in the very last stages of magma ascent towards the surface (level 3 in Fig. 8). Interestingly, these timescales are similar to diffusion timescales recorded in tektite glasses^70^.

These timescales are different from timescales obtained from diffusion in minerals^71^. For example, olivine Fe–Mg diffusion modelling returns timescales of < 4 days for xenolith transport to the surface^72^ and 2–45 days for phenocryst rim zonations in the 1949 eruption^32^. These differences are expected, as elemental diffusion in minerals is several orders of magnitude slower than in melts. Hence, diffusion of most elements in minerals (e.g. Fe–Mg diffusion in olivine) cannot record mixing processes occurring in the very shallow conduit (as is the case in this work), but rather register processes occurring at depth in the system, and therefore much longer timescales.

The close textural relationship observed between melt heterogeneities and vesicles suggests a connection between bubble growth/migration and melt mixing. Filament morphologies in the La Palma tephra are remarkably similar to those produced in basalt-rhyolite bubble transfer experiments^73^, suggesting that bubble nucleation and migration in a heterogeneous magma during ascent could have enhanced mingling and mixing of the two melt components by dragging basanitic melt tails while moving into the tephritic melt. Analytic profiles in our natural system also resemble experimental profiles^73^, with plateau shapes, multiple peaks and bell shapes (Fig. 5). Such complex profile shapes indicate more than one stage of melt injection and/or stretching^73^. Sectioning effects in these tails can explain the presence of detached filaments and blobs apparently not associated to bubbles (see Fig. 3 in Wiesmaier et al*.*^73^). Interestingly, Wiesmaier et al. also report the occurrence of melt tails attached to vesicles in natural basalts from Axial Seamount, which resemble some of the structures observed in this work. Heterogeneous bubble nucleation could potentially have had a role in a heterogenous La Palma 2021 magma, both at the melt-melt interface and in mineral surfaces. The presence of magnetite crystals inside some vesicles (Fig. 4f) suggests that magnetite could have played a role as favourable bubble nucleation site^19,74^.

Another possible reason for the very short timescales recorded by the filamentary structures are turbulences during magma ascent, which strongly enhance mingling^49,67^. Turbulent conditions during ascent in a dyke are facilitated by the low viscosity of basanitic magma^41^, even at low ascent rates^75^. At such conditions bubble rise can be subordinate for mixing. However, bubble-induced mixing should become dominant at the very last ascent stage, when magma is fragmented by large bubbles rising at high velocities to produce strombolian explosions or lava fountains. This is the likely scenario where the short-lived filamentary structures can be generated.

Given the low volume ratio of basanitic to tephritic glasses in the filament-rich areas and their large compositional contrast, an important issue is the provenance of these primitive melts and their survival up to shallow levels in the plumbing system, where the filamentary structures were produced. The markedly bimodal distribution of groundmass glass composition in September 23 samples (Fig. 3) points to incomplete mixing during ascent through the crust. The presence of more primitive mafic compositions in the filamentary structures would require the presence of larger volumes of basanite melt, ascending mostly undisrupted (i.e., without major mixing) up to very shallow levels where disruption and mixing occur. An apparent bimodality in whole rock data during the first week of the eruption^24^ could be evidence of these batches, and additional insight could be obtained from glass analysis in dense time-series sample collections. Thus, the observed filamentary structures likely represent the last stage of a continuous mingling process during magma ascent, with previous melt stretching and folding events dissipating quickly during ascent through the interface of both magmas in a very dynamic environment. The scenario depicted here resembles that of the 1949 eruption where tephrite and basanite magmas showed limited mixing, in the form of dark bands (*schlieren*), during the transition from one lithology to another^32^. Fe–Mg diffusion modelling in olivine from this eruption suggests that such longer mixing process could have started between 2 and 45 days before the eruption^32^. Although no olivine diffusion timescales have been published for the 2021 eruption so far, those from 1949 would be consistent with a larger mixing event occurring a few weeks before the eruption onset. Melting of old basanite from the volcanic edifice can be ruled out as an explanation for the filaments, due to the very high temperatures needed to obtain almost complete melting needed to produce the observed compositions.

### Magma sources and ascent

From the evidence obtained in this work, a picture of the plumbing system under La Palma during the 2021 eruption can be sketched (Fig. 8). The occurrence of heterogeneous glasses in the early stages of the eruption and the overall chemical distribution of groundmass glasses, coupled to the varying phenocryst cargo in lavas, attest the participation of different magma batches in the eruptive dynamics. The results of clinopyroxene thermobarometry suggest a deep reservoir at ca. 25–35 km b.s.l. (with alternative values of 21–27 km from the MAM^60^ model), which might be the source of the more primitive basanitic magmas, while kaersutite barometry in tephrites may point to two separate storage levels at 16–23 and 24–29 km b.s.l.. Clinopyroxene rims and cores from the tephrites record the same pressure interval as those from late basanites, raising the possibility of a similar storage level for both magma types. This scenario strongly resembles that proposed for the CV 1949 eruption, where basanites and tephrites were found to have similar storage depths^32^. During the early eruption stage, the first basanite magmas arriving from the deep reservoir interacted with the crystal-rich, kaersutite-bearing tephritic magma. The disappearance of amphibole after ca. 1 week and the evolution of both groundmass glass and whole rocks towards more primitive compositions^24,46^ indicate that the tephritic reservoir did not contribute to erupted products in October-December. The 10-h long eruptive hiatus on September 27th likely marked the last contribution of the tephritic magma to erupted materials.

The early eruptive stage dominated by tephritic magma is coincident with Stage 1 proposed by del Fresno et al.^38^, based on a thorough study of coeruptive seismicity. They suggest that a “mushy” reservoir may have been present at a depth of ca. 12 km, potentially representing the source of the early-stage tephritic lavas. This depth is the origin of the seismic swarm starting on 11 September^36,38^, suggesting that this date may be a probable onset of the tephrite-basanite interaction. In previous eruptions, olivine diffusion recorded pre-eruptive timescales overlapping with this date^32^. However, kaersutite barometry suggests a depth of 16 to 23 km for tephrite storage (with due caution due to the large errors inherent to amphibole-based barometry^63,76^), and the storage pressure of clinopyroxenes in the tephrite also argues for a deeper level. Thus, it is unclear whether the pre-eruptive 12 km seismic swarm is the expression of a protracted storage region or reflects a short-term magmatic storage level at the base of the oceanic crust^33^ before initial dike propagation. Furthermore, kaersutite cores register depths of 24–29 km, which are within the upper pressure interval for kaersutite cumulate xenoliths erupted during the CV 1971 Teneguía eruption^34^. Overall, these data suggests that tephritic melts followed a polybaric, long-term evolution before the eruption, and were only reactivated days before eruption onset by mixing with basanitic magmas.

The deeper reservoir, centred at 26–33 km according to Putirka^59^ clinopyroxene barometry, agrees well with the location of pre-eruptive seismicity recorded between 2017 and 2021^35,36,38^, and perhaps up to 2010^39^. It also overlaps with the depth range inferred for early clinopyroxene crystallization in the 1949 and the 1971 eruptions^32,34^, and adjoins the depth range for late clinopyroxene crystallization of historic and prehistoric CV magmas (15–26 km)^32,33^. The latter range also overlaps with our results from the MAM^60^ calibration, and with pressures derived from CO_2_ density measurements in olivine-hosted fluid inclusions^77^, suggesting last equilibration depths of 15 to 27 km. We note, however, that the comparison of petrological data with pre- and syn-eruptive seismic data is not unambiguous. Depending on the velocity model used, the two clusters of syn-eruptive seismicity in 2021 were located either at 10–14 and 33–39 km depth^38^, or at 7–12 and 20–25 km depth^37^. Hence, the inferred depth range for late clinopyroxene crystallization would be located either between both seismic clusters in a temporarily aseismic zone or would perfectly overlap with the deep cluster. In consequence, more detailed work is necessary to define source depths of the intervening magmas.

### Conclusion

The 2021 Tajogaite eruption of the Cumbre Vieja edifice, La Palma, provides an excellent opportunity to observe pre- and syn-eruptive processes with an unprecedented level of detail. This work highlights the rare occurrence of compositionally heterogeneous melts in glassy tephra clasts erupted in the fifth eruption day, during a period of enhanced explosive activity. Micrometer-size filaments of primitive Si-poor and Fe, Mg-rich basanitic melt are associated to bubbles embedded in a more evolved glass, suggesting a link between melt mingling and bubble growth and migration. Diffusion modelling supports the idea that these features are very short-lived, in the order of seconds to a minute. They can therefore be related to the latest phase of magma ascent, in the shallow conduit or even in the lava fountain. The filaments are interpreted as products of late stage mingling between tephritic and basanitic melts enhanced by bubble transfer during the turbulent ascent of a heterogeneous magma, rising from sub-Moho depths. Our study provides a new insight on the latest phase of magma ascent in the CV 2021 eruption and indicates a possible link between magma mixing, degassing and enhanced explosivity. Subtle filamentary structures that testify incomplete mixing are entirely obliterated in crystallized lava flow groundmass, making a detailed study of pyroclastic material mandatory to resolve the fast evolution of magmatic processes during an eruption.

## Methods

### Sampling

The materials used in this study were sampled both during the eruption by author T.B. on 23 September 2021 and by A.K. in October; and during a sampling campaign conducted by T.B. and D.G.-G. between 24 and 26 March 2022. A selection of 6 lava samples, for which emplacement date is accurately known, was collected in flow lobes emplaced throughout the eruption. These included the tephritic lava flow emplaced on 23 September at the base of Montaña de Cogote (samples CV-2309 and LP2021-1); and four samples from basanitic lavas emplaced on 13 and 21 October; and 23 and 29 November (LP2021-2, CV-2110, CV-2311 and CV-2911; Fig. 1c). Syneruptive tephra collection was carried out in the Las Manchas cemetery and El Paraiso. The Las Manchas sample (LM-2309) consisted in fine tephra collected during a period of strong strombolian explosions (22:45–23:15 local time) with conspicuous atmospheric shock waves. The El Paraiso sample (PAR-2309) consisted in coarser lapilli-sized tephra. Two additional tephra samples were collected in the pyroclastic deposits during the sampling campaign. Sample CV21P-B was sampled in a thick lapilli layer in Las Manchas (Fig. 1c). This layer corresponds to unit LU 2.3 of Romero et al.^40^ and was emplaced between 26 September and 10 October 2021. Sample CV21P-A was collected in a proximal location, immediately to the west of the main cone. It corresponds to the latest explosive pulses of the eruption and was emplaced on 1–2 December 2021. This sample includes a dense bomb from the same eruptive pulse (CV21P-DB). Further information on collected samples is available in the Supplementary Information.

### Electron microprobe

Major and minor element concentrations were determined in groundmass glass and minerals using a Cameca SX-100 electron microprobe (EPMA) at the Department of Geosciences of the University of Bremen. Glassy groundmass was analysed in tephra samples from 23 September, 26 September to 10 October and 1–2 December. Glasses were analysed using an acceleration voltage of 15 kV, beam current of 10 nA, and defocused beams of 5 µm for areas with inhomogeneous glass, and 10 µm for general groundmass glass. Pyroxene, amphibole, olivine and oxides were analysed using an acceleration voltage of 15 kV and beam current of 10 nA with focused beam. Counting times were 20 s on peak for most elements. Standard materials were analysed both at the beginning and end of each analytical session for precision and accuracy control; the data are given in the Supplementary Information. These standards include basaltic glasses VG-2 and VG-A99, Kakanui augite (NMNH 122142), Cr-augite (NMNH 164905), Kakanui hornblende (NMNH 143965), San Carlos olivine (NMNH 111312-44), chromite (NMNH 11075) and ilmenite (NMNH 96189). For concentrations > 1 wt% accuracy is better than 3% in most cases: the data are given in the Supplementary Information.

Three high-resolution profiles were also obtained with the beam profile modality in the electron microprobe, by keeping the sample stationary and sweeping the beam in a 25 µm line. In this modality, analytical conditions were: beam diameter of 2 µm, acceleration voltage of 15 kV, beam current of 40 nA and a dwell time of 1 s. per point, for a total of 100 points per profile. The elements analysed were Si, Ti, Al, Fe, Mg, and Ca. Alkalis (Na and K) were analyzed but showed some loss and were thus excluded from the final dataset. Castaing approximation was used to transform intensities to oxide wt% and the obtained concentrations were corrected based on quantitative analyses at discrete spots along or close to the profiles. Analytical precision of this approach was 3% or better except for Ti (around 5%) and P (around 8%).

### Scanning electron microscope (SEM)

A Scanning Electron Microscope (SEM) was used to obtain high-resolution back-scattered electron (BSE) and wavelength-dispersive X-ray spectroscopy (WDS) maps in tephra and lava samples. BSE images were acquired using a JEOL JSM-7610F gun emission scanning electron microscope installed at the Institut für Mineralogie of the Leibniz Universität Hannover, Germany, using an accelerating voltage of 15 kV and a working distance of 15 mm. Bruker ESPRIT software was used for image and map acquisition.

### Laser ablation ICP-MS

The major element concentrations of the crystalline groundmass of three lava samples (LP2021-1, LP2021-2, CV-2911) and dense bomb CV21-DB were determined by laser ablation inductively-coupled plasma-mass spectrometry (LA-ICP-MS) at the University of Bremen with a NewWave UP193ss laser coupled to a ThermoElement2 mass spectrometer, using the method described in Klügel et al.^31^. A laser beam with a diameter of 100 µm was rastered across the groundmass for 60–180 s for each analysis. Final values for each sample are the average of four to eight raster analyses. Analysed isotopes were ^23^Na, ^24^Mg, ^27^Al, ^28^Si, ^31^P, ^39^K, ^48^Ti, ^55^Mn and ^56^Fe. Ca was used as internal standard. USGS glass BCR-2G was used as external calibration standard, and BHVO-2G was employed for accuracy control^78^. Data for samples and reference materials are given in the Supplementary Information.

### Thermobarometry

Clinopyroxene-melt equilibria were evaluated using the barometer of Eq. (31) and the thermometer of Eq. (33) of Putirka^59^. The standard error of estimate (SEE) for these calibrations is ± 290 MPa and ± 42 °C, respectively, but we note that errors can be substantially lower if several pyroxenes are analysed and the estimates averaged^79^. Additionally, the thermobarometer for mafic alkaline melts of Mollo et al.^60^ was applied, with SEE of ± 150 MPa and ± 20 °C. These models were chosen for consistency reasons and based on their suitability for H_2_O-bearing basaltic melts. Final pressures and temperatures were obtained through an iterative process^80^. In a first step, pressure and temperature were calculated for each mineral-melt pair, and averages were obtained for each rock sample. In subsequent steps, the average pressure was used as input in the thermometer equation, and the average temperature was introduced in the barometer equation, until the results converged. Simultaneously, equilibrium parameters were monitored and pairs falling out of equilibrium were discarded using the following filters: (1) the Fe–Mg partition coefficient between mineral and melt (*K*_*D*_(Fe–Mg)^*cpx-mel*t^) had to be 0.28 ± 0.08^59^; and (2) error margins between measured and calculated DiHd (diopside-hedenbergite), CaTs (calcium-Tschermak) and EnFs (enstatite-ferrosilite) components were set at 0.10, 0.06 and 0.05, respectively (cf.^59^). For DiHd, a T-dependent calculation^81^ was used. Pyroxene analyses were paired to the average pyroclast glass composition from roughly the same eruptive period, with a melt H_2_O concentration of 1.1 wt%^82^.

Crystallization P–T conditions were estimated from kaersutites by using the amphibole-only calibration of Ridolfi and Renzulli^61,62^. The uncertainties on the determined P and T values is estimated by the authors to be ± 12% and ± 22 °C, respectively, although P errors can be substantially larger^63,76^.

Obtained pressures were converted into depths by using a three-layered model including the island edifice, ocean crust and lithospheric mantle, with respective densities of 2700, 2980 and 3400 km/m^3^. The depths of the island edifice and Moho were set at 4.5 and 12.5 km^83^, respectively.

### Diffusion modelling

To obtain a first-order constraint on timescales over which the filamentary structures in heterogeneous areas could survive at high temperatures, an attempt of modelling the diffusive transport in the compositional profiles from mixing areas was made, without considering the effects of advection. The diffusion equation was solved using a finite difference numerical model, which was applied to the diffusion of Al_2_O_3_, FeO, and CaO, elements with published diffusion data and not apparently affected by multicomponent effects (Fig. 5). The equation of the finite difference model in an open system is:

1 $${C}_{i,j+1}={C}_{i,j}+\frac{D\times \Delta t}{\Delta {x}^{2}}\left({C}_{i+1,j}-2{C}_{i,j}+{C}_{1-1,j}\right).$$

where *C*_*i,j*_ is the concentration at a position *i* in the profile and time step *j*. Δ*x* and Δ*t* are the distance and time steps, respectively. Δ*x* is equal to the distance spacing in the microprobe profiles, and *Δt* must be chosen so that the term $\frac{D\times \Delta t}{\Delta {x}^{2}}<0.5$, for computational stability reasons.

The measured concentration profiles were used as initial concentration distribution, and their evolution with time was calculated. Owing to rapid homogenization, analytical uncertainty did not significantly affect the results. To avoid boundary problems, models were extended for 25 µm to each side of the modelled profile. In each model step, the normalized concentration variance^67^, ${\sigma }_{n}^{2}$ was calculated, to monitor the degree of diffusive homogenization of the profile.

However, most published diffusion data belong to supra-solidus conditions, with very few diffusivities at temperatures close to those assumed for natural plumbing systems. Among them, Neave et al.^10^ have recently provided diffusion coefficients (*D*) for major elements in crystal-bearing basalt with 1 wt% H_2_O at 1190 °C. Such diffusivities are consistent with the Arrhenius relations observed at higher temperatures^50^ and provide a reasonable starting point for timescale estimations in natural magmatic systems. Arrhenian parameters^10^ were used to calculate diffusivities at 1055 °C (Supplementary Table 2). This temperature reflects crystallization temperatures in the tephritic reservoir, as indicated by amphibole thermobarometry, and therefore represents a minimum temperature estimate of the system during basanite–tephrite interaction. Higher temperatures would increase diffusion rates and decrease associated timescales.

A further complication in the modelling of diffusive timescales in natural melts is the effect of uphill diffusion, arising from interelement cross-diffusivities in a multicomponent system^84–86^. In our dataset, uphill diffusion is only evident in the MgO data in profiles B and C (Fig. 5) where distinct minima in composition are observed at both sides of the filaments (red arrows in Fig. 5). Consequently, MgO was not considered in the diffusion modelling.

### Supplementary Information

Supplementary Information 1.

Supplementary Information 2.

Supplementary Information 3.

Supplementary Information 4.

Supplementary Information 5.

Supplementary Information 6.

## Data Availability

All data generated or analysed during this study are included in this published article and its Supplementary Information files.
