# Supplementary material for: Bubble-enhanced basanite–tephrite mixing in the early stages of the Cumbre Vieja 2021 eruption, La Palma, Canary Islands
Source: Sci Rep. 2023 Sep 8;13:14839. doi: 10.1038/s41598-023-41595-3 (PMC10491805; doi:10.1038/s41598-023-41595-3)
Supplement: Supplementary file 1 — Supplementary Information 1. [file 41598_2023_41595_MOESM1_ESM.pdf]

SUPPLEMENTARY INFORMATION

Bubble-enhanced basanite-tephrite mixing in the early stages of the Cumbre Vieja 2021 eruption, La Palma, Canary Islands

Diego González-García[1], Thomas Boulesteix[2], Andreas Klügel[3], François Holtz[1]

[1] Institut für Mineralogie, Leibniz Universität Hannover, Germany

[2] Department of Earth and Life Sciences, Instituto de Productos Naturales y Agrobiología (IPNA-CSIC), San Cristóbal de La Laguna, Tenerife, Spain

[3] Fachbereich Geowissenschaften, Universität Bremen, Germany

LIST OF MATERIALS

Supplementary Information 1. Supplementary figures and tables (this file)

Supplementary Information 2. Sample information.

Supplementary Information 3. Electron microprobe dataset (glass and mineral, including standards)

Supplementary Information 4. Groundmass LA-ICP-MS data (including standards)

Supplementary Information 5. Thermobarometry

Supplementary Information 6. Diffusion models

SUPPLEMENTARY FIGURES

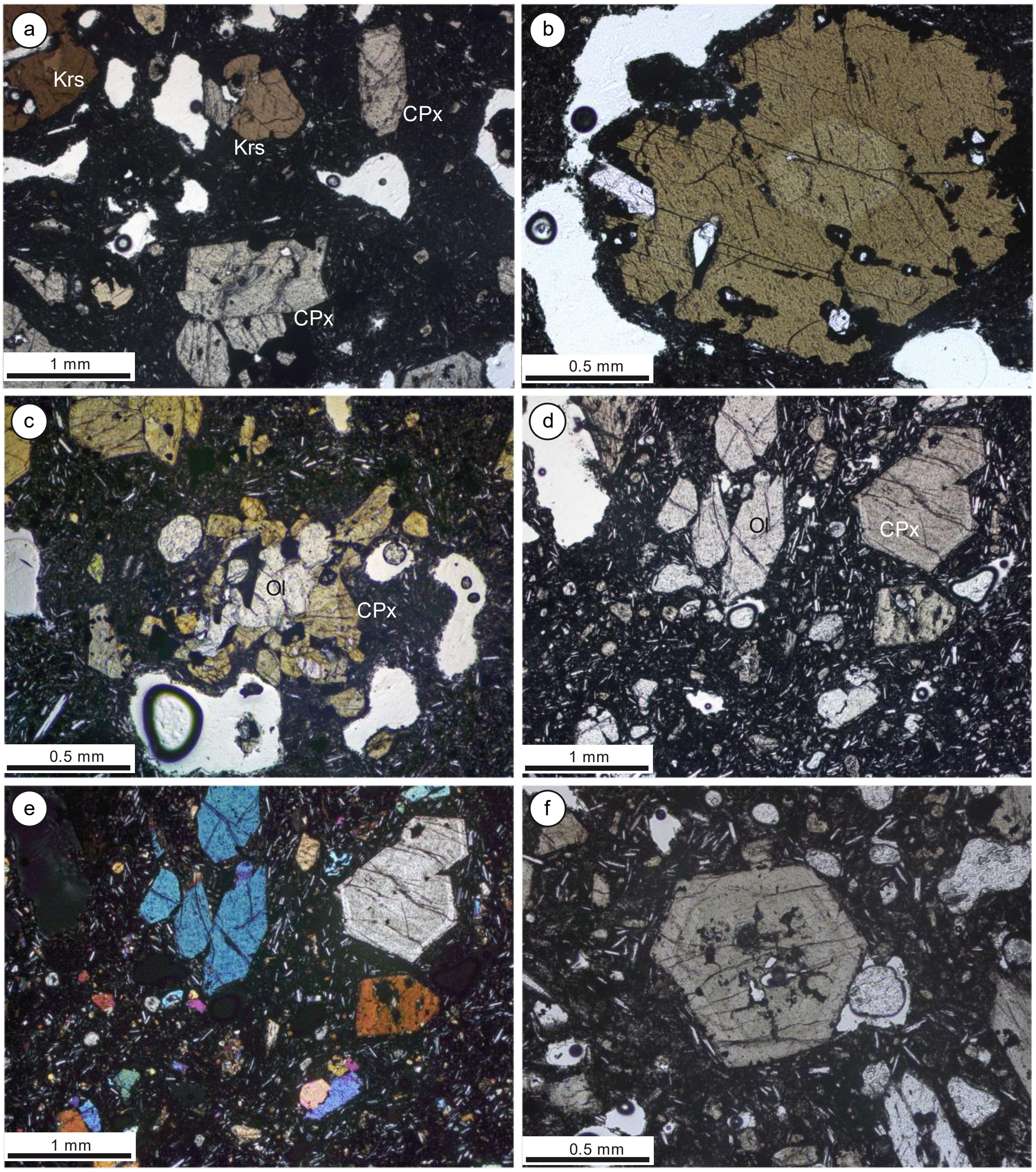

Supplementary Figure 1: Petrographic aspects of the Cumbre Vieja 2021 tephritic (a-c) and basanitic (d-f) lavas. (a) Kaersutite (Krs) and clinopyroxene (CPx) phenocrysts in a tephrite lava from September 23, 2021. (b) Zoned kaersutite in tephrite. (c) Cumulate xenolith composed of olivine (Ol) and CPx. (d) General aspect of a basanite sample from October 21, 2021, dominated CPx and Ol phenocrysts. (e) Sample image in crossed nicols. (f) Zoned CPx phenocrysts with a resorbed core.

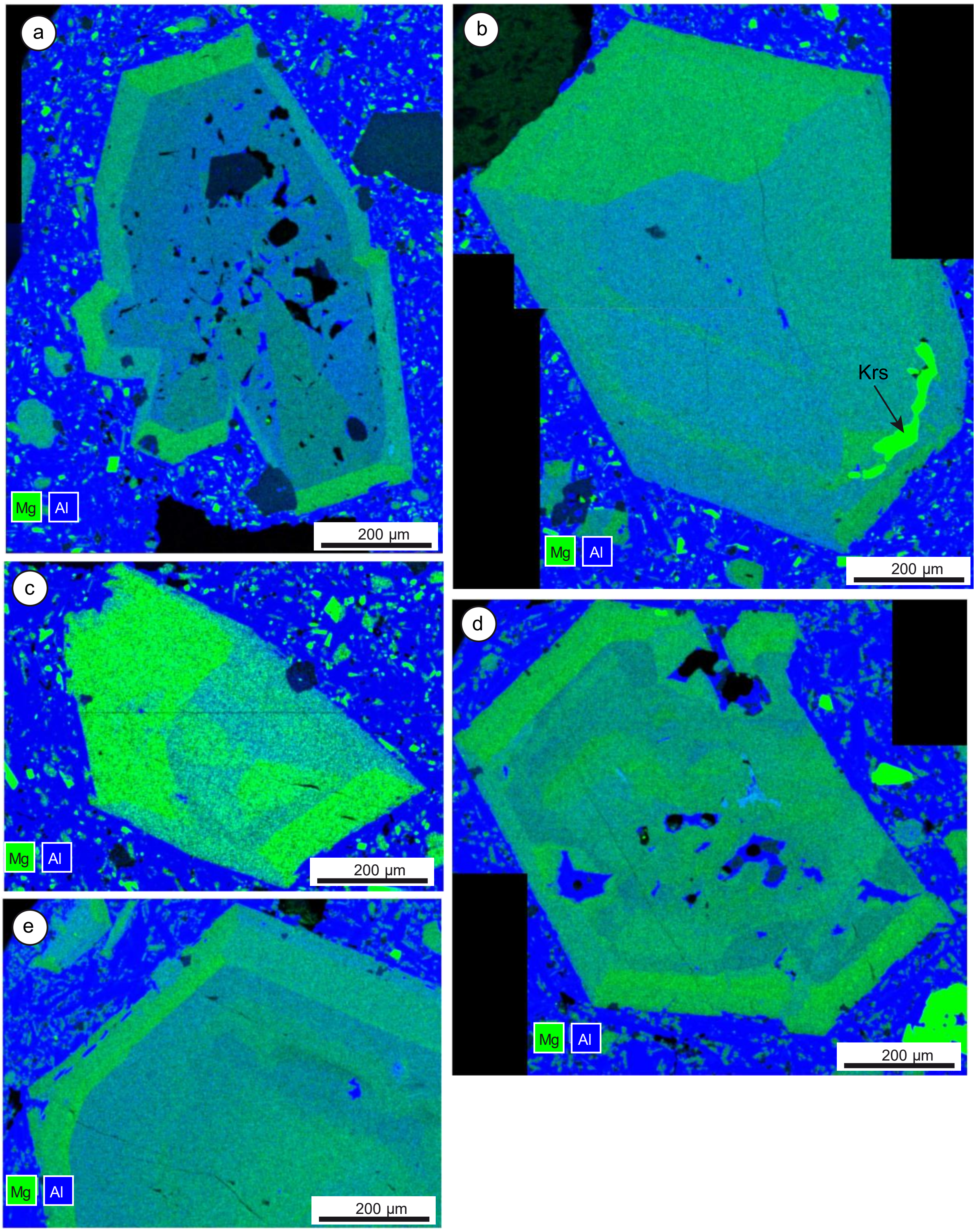

Supplementary Figure 2: SEM EDX maps showing Mg-Al zoning of clinopyroxene phenocrysts from tephrite samples CV-2309 (a-c) and basanite CV-2110 (d-e), emplaced respectively on 23 September and 21 October 2021.

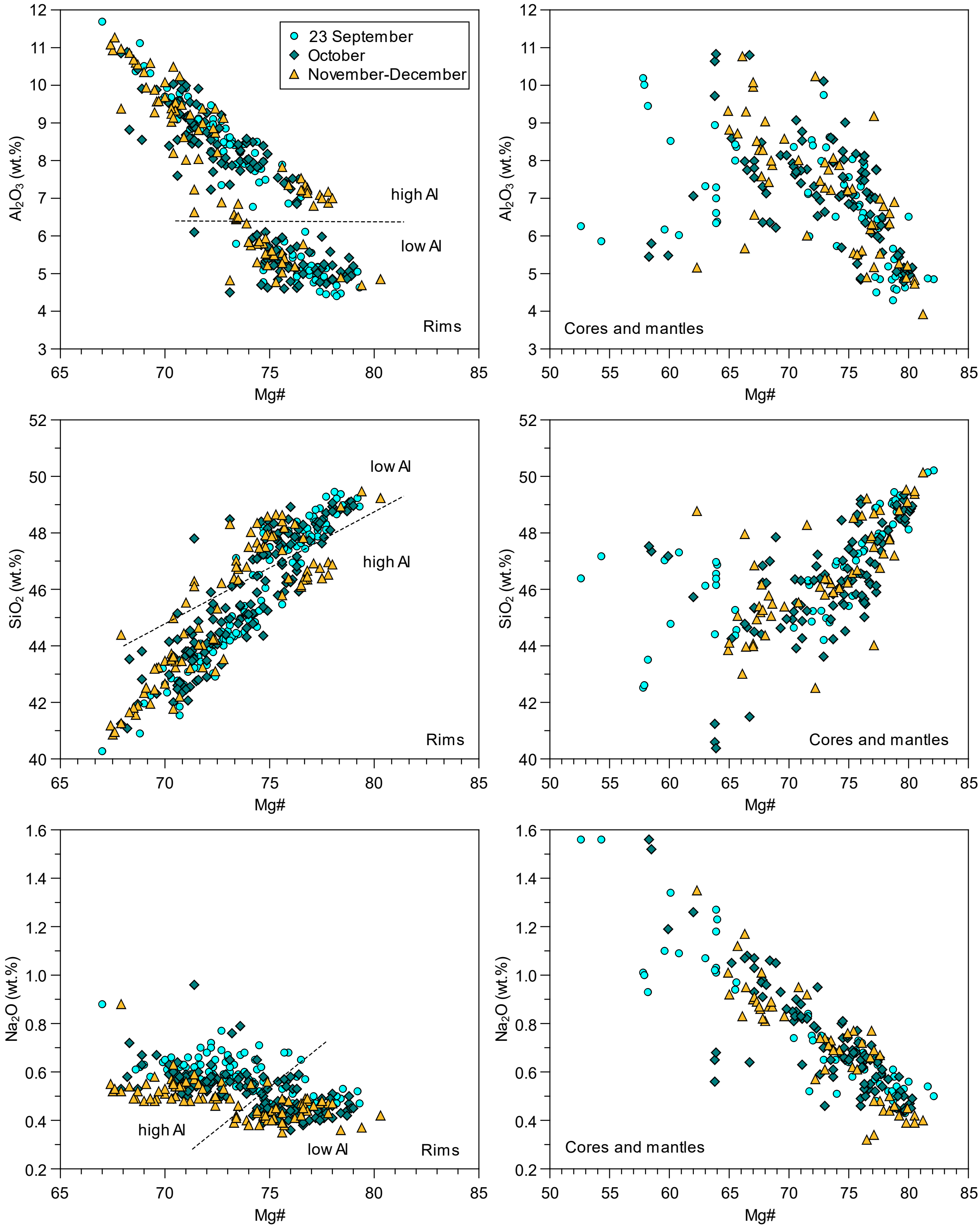

Supplementary Figure 3: Chemical compositions of clinopyroxene rims (left column) and interiors (right column) from both lavas and tephras erupted throughout the eruption. Sector zoning is evident in rims, but much less in core analyses, which also have a wider compositional span.

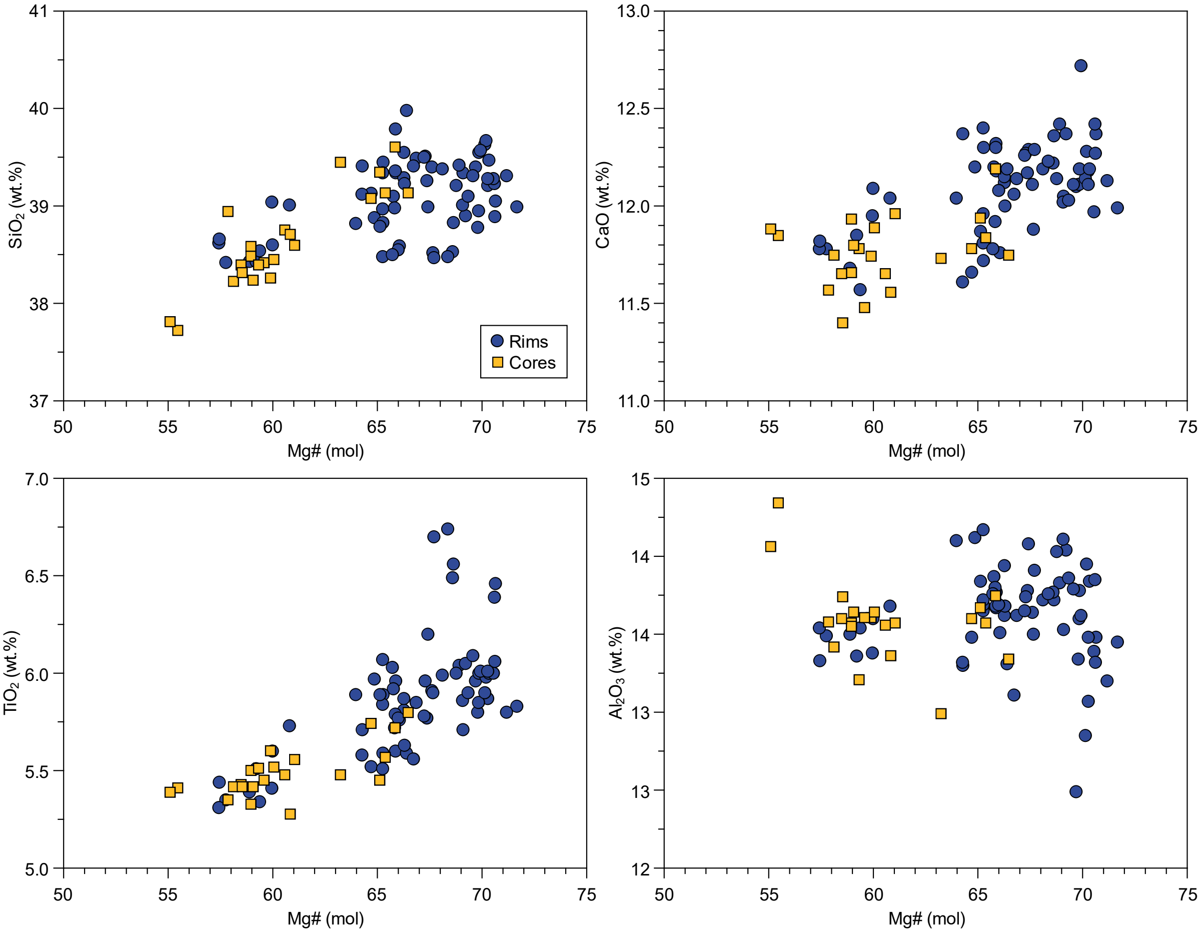

Supplementary Figure 4: Chemical compositions of kaersutite cores and rims in tephritic lava and tephra samples from on September 23, 2021 (eruption day 4).

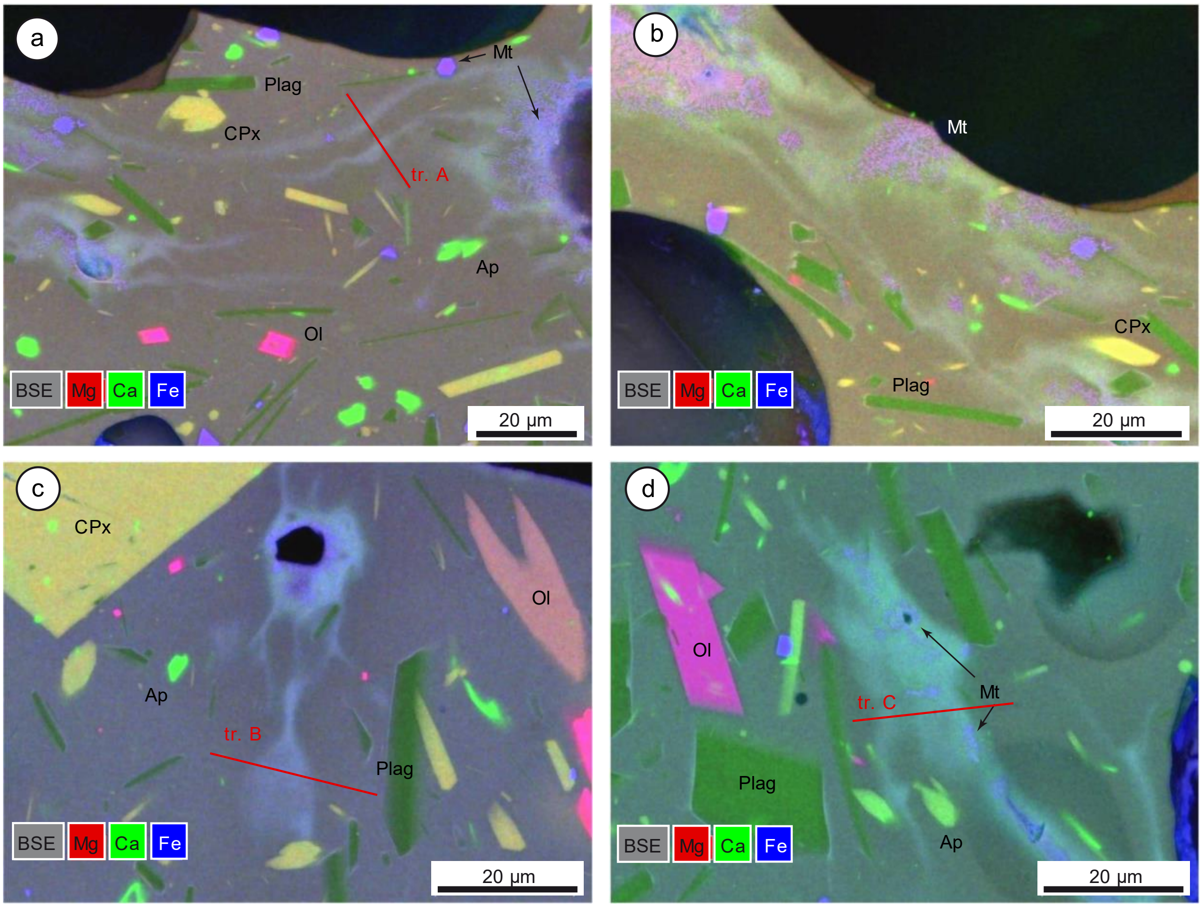

Supplementary figure 5. SEM compositional maps showing a detail of the mixing areas shown in **Figure 3a**. Transects A, B and C are marked. Note the formation of dendritic magnetite at bubble rims, very probably related to melt degassing at the bubble rim. Plag: plagioclase; CPx: clinopyroxene; Ol: olivine; Ap: apatite; Mt: titanomagnetite.

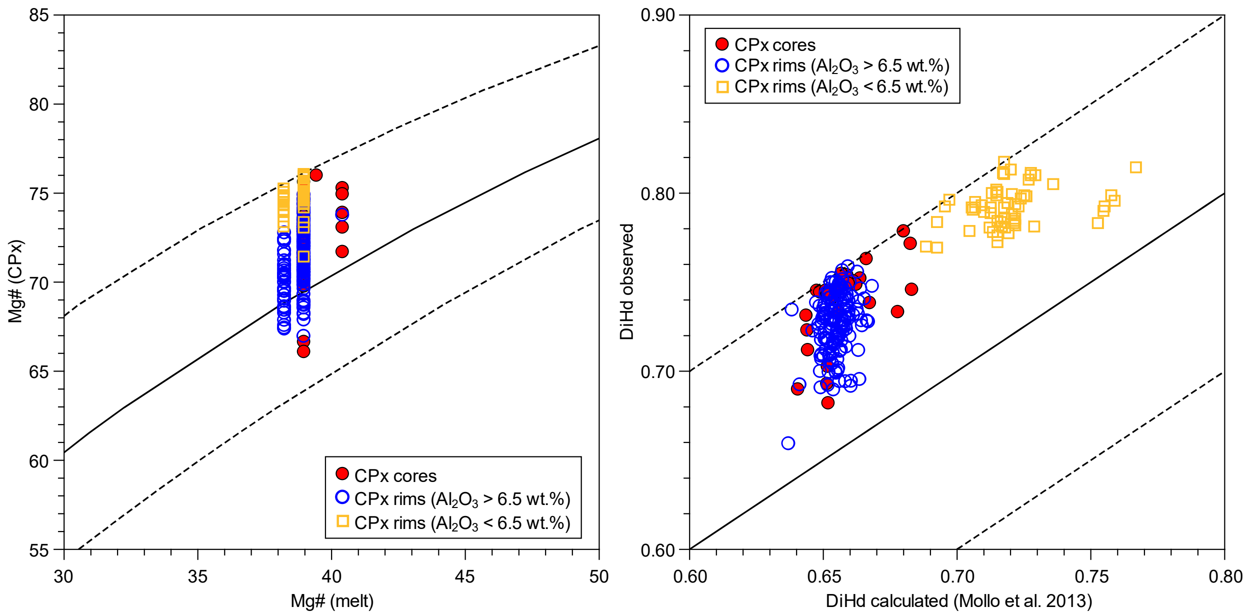

Supplementary Figure 6: Equilibrium tests for clinopyroxenes (CPx). (a) Fe-Mg exchange diagram, with $k_D(Fe\text{-}Mg)^{CPx\text{-}liq}$ = 0.28 ± 0.08 (Putirka, 2008). (b) Calculated vs predicted DiHd (diopside-hedenbergite), with calculated values from the T-dependent calculation of Mollo et al. (2013).

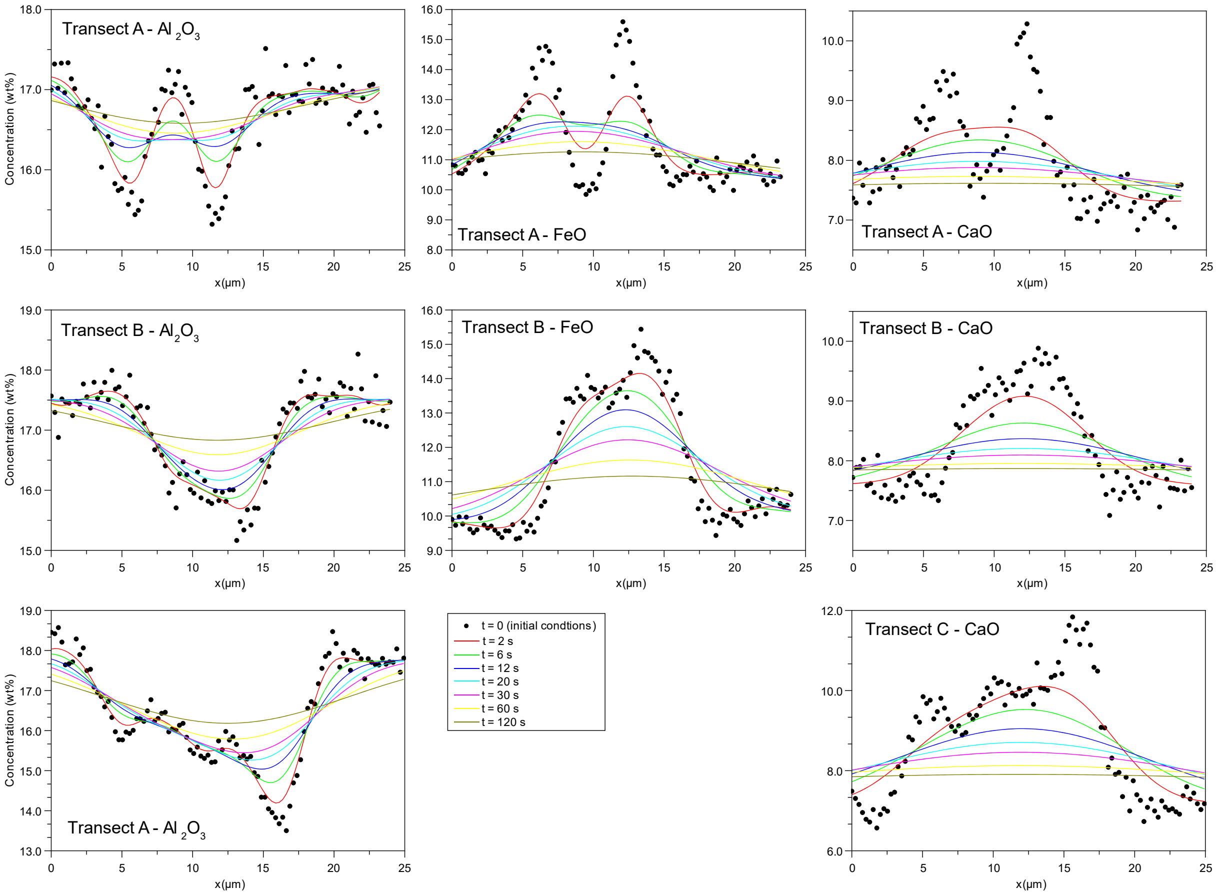

Supplementary Figure 7: Results of diffusion modelling of Al, Fe and Ca in the three EPMA beam transacts cutting across filamentary structures. For details about the model setup, please refer to the main text.

SUPPLEMENTARY TABLES

Calibration	Mineral	n	P	sd	T	sd	Depth (km)
Ridolfi & Renzulli, 2012	Krs cores	22	783	46	1065	8	24-29
Ridolfi, 2021	Krs rims & mantles	68	581	44	1052	13	16-23
	CPx high-Al rims	149	908	43	1134	4	26-33
Putirka, 2008	CPX low-Al rims	57	451	166	1100	13	0-25
	CPx cores	27	888	125	1137	13	25-35
	CPx high-Al rims	185	753	35	1147	9	21-27
Mollo et al., 2018	CPX low-Al rims	36	544	106	1104	22	6-22
	CPx cores	48	726	63	1139	15	18-27

Supplementary Table 1: Summary of thermobarometric estimations using Kaersutites (Krs) and clinopyroxenes (CPx). n = number of mineral or mineral-melt pairs. Pressures have been converted to depths assuming a three-layer model with densities of 2750, 2980 and 3400 kg/m^3 for island edifice, oceanic crust and mantle, respectively, and depths of 4.5 km and 12.5 km for ocean floor and Moho, respectively. Low-Al CPx rims likely do not represent real storage conditions, but instead result from decompression effects in CPx.

Component	log D_0 (m^2/s)	E_a (kJ/mol)	log$D_{1055°C}$ (m^2/s)
Al	-3.04	245	-12.67
Fe	-3.64	219	-12.26
Ca	-3.95	195	-11.62
Na	-2.39	216	-10.88

Supplementary Table 2: Arrhenius parameters (Neave et al., 2021) and resulting diffusion coefficients of Al, Fe, Ca and Na in basalt at 1 wt.% H_2O, calculated for a temperature of 1055°C. Fast diffusing Na is not used in the modelling but is shown for reference.
